# Do-gooder derogation in children: the social costs of generosity

**DOI:** 10.3389/fpsyg.2015.01036

**Published:** 2015-07-21

**Authors:** Arber Tasimi, Amy Dominguez, Karen Wynn

**Affiliations:** Department of Psychology, Yale University, New Haven, CT, USA

**Keywords:** morality, social cognition, cognitive development, prosocial behavior, social comparison

## Abstract

Generosity is greatly valued and admired, but can it sometimes be unappealing? The current study investigated 8- to 10-year-old children’s (N = 128) preference for generous individuals, and the effects of social comparison on their preferences. In Experiment 1, children showed a strong preference for a generous to a stingy child; however, this preference was significantly reduced in a situation that afforded children a comparison of their own (lesser) generosity to that of another child. In Experiment 2, children’s liking for a generous individual was not reduced when that individual was an adult, suggesting that similarity in age influences whether a child engages in social comparison. These findings indicate that, by middle childhood, coming up short in comparison with a peer can decrease one’s liking for a generous individual.

## Introduction

From the first few months of life, humans are attracted to those who behave kindly toward others (Hamlin et al., 2007, 2010; Hamlin and Wynn, 2011). Before their second birthday, young toddlers will even reward individuals for their positive behaviors (Hamlin et al., 2011). Such an attraction toward prosocial individuals is robust across development. For example, young children help individuals who engage in positive behaviors more than those who engage in negative ones (Vaish et al., 2010). In adolescence, individuals who behave prosocially are more likely to be accepted into peer groups (Parkhurst and Asher, 1992). Among adults, prosocial individuals hold a high social status and are sought as interaction partners (Hardy and Van Vugt, 2006). Beyond the laboratory as well, everyday life abounds with examples of positive behaviors being lauded, from elementary schools that award students certificates when they are “caught being good” to news reports that praise philanthropists for their charitable work.

Despite this strong favoritism toward benevolent others, a number of recent studies suggest that acting too generously can sometimes be off-putting, a phenomenon often referred to as “do-gooder derogation.” For example, recent work finds that in some contexts adults dislike the extremely generous: When evaluating players who contributed various amounts to a group effort, adults expelled over-contributing individuals as much as under-contributing ones (Parks and Stone, 2010). Dislike of do-gooders is not unusual; some evidence suggests that it may be universal (Herrmann et al., 2008). In particular, in a public goods game, where participants choose how much to donate to a public pot (of which the total is then multiplied, and subsequently divided equally among players), individuals from a range of cultures punished high contributors as much as low contributors.

What could explain this aversion to generous individuals? People may resent others’ generosity for reasons involving social comparison (Monin, 2007). In particular, people are more likely to reject those who do the right thing in situations that evoke social comparisons with others (Monin et al., 2008). For example, participants who took part in a task perceived as racist (e.g., asked to identify the likely burglar in a suspect lineup after being given information that pointed to the lone African American in the lineup) subsequently rejected an individual who refused to go along because the task was “offensive,” whereas mere observers embraced him. A popular example of this aversion also comes from meat eaters who use negative words to describe vegetarians because they believe that vegetarians feel morally superior (Minson and Monin, 2011). In fact, people asked to taste meat are more likely to dislike an individual who refuses to do so, if that refusal is for moral rather than non-moral reasons (Cramwinckel et al., 2013). These findings show that adults often tend to feel negatively toward individuals who morally outshine them.

Indeed, previous theorizing suggests that the tendency to engage in social comparison is a fundamental aspect of everyday social life (Festinger, 1954; Fiske, 2011). A growing body of research has shown that comparing oneself to others is an early-emerging dimension of social cognition (e.g., Ruble et al., 1994; Pomerantz et al., 1995; Rhodes and Brickman, 2008). For example, 5- and 6-year-olds willingly incur a personal cost to ensure that they receive more resources than another child (Sheskin et al., 2014). Moreover, 7- to 13-year-olds feel more bitter upon failing a speeded reaction time task after learning that another child succeeded (Steinbeis and Singer, 2013). Although this literature has demonstrated that childhood is a period rife with social comparisons (for review, see Dijkstra et al., 2008), very little is known about whether children are affected by another’s generous behavior relative to their own.

The current study examined whether children reject individuals whom they see as more generous than themselves. Generosity is considered one of the most important spiritual values in the Bible (Corinthians 1:13), and it is admired universally, and from very early in life; for example, even young infants prefer an individual that gives rather than takes (Hamlin and Wynn, 2011). Thus, understanding whether children reject individuals on the basis of their generosity provides a strong test of the role of social comparison in do-gooder derogation, especially because previous work on this topic has focused on ambiguously “moral” domains such as vegetarianism (Minson and Monin, 2011; Cramwinckel et al., 2013) and “political correctness” (Monin et al., 2008).

We focused on children aged 8–10 to investigate whether appearing selfish *relative* to another individual influences their social preferences. From the ages of 7–8 onwards, children select equitable resource distributions between themselves and another child, while younger children select distributions that favor themselves (Fehr et al., 2008; Sheskin et al., 2014). These results suggest that, by 7–8 years of age, children are especially concerned with not being—or not appearing—selfish. In Experiment 1, we assessed whether children would be less likely to prefer a generous child who behaves more generously than themselves. In Experiment 2, we ran a stronger test of our social comparison hypothesis: because social comparisons are strongest for those most similar to ourselves (Goethals and Darley, 1977; Wood, 1989; Suls et al., 2002), we assessed whether children’s liking for a more generous individual was influenced when that individual was an adult, rather than another child. This manipulation decreases the potential for social comparison and, thus, children should reliably prefer a generous adult, regardless of whether children first give themselves.

## Experiment 1

### Participants

Sixty-four children (40 girls; mean age = 9.26 years; range = 8.40–10.41 years) participated in the study. Two additional children were excluded due to experimenter error. Children were recruited from public schools in the greater New Haven, Connecticut area and tested individually in a quiet room at their elementary school. Parents of participating children gave written informed consent; children also provided oral assent. Data were not gathered on participants’ race/ethnicity; however, children were tested in schools serving communities that were primarily white and middle class. The Human Subjects Committee at Yale University approved all study procedures.

### Procedure

Children were given six stickers and randomly assigned to the *Comparison* (*N* = 32) or *No Comparison* (*N* = 32) condition. In the *Comparison* condition, the experimenter showed children a photograph of a child, Gary, telling them Gary had no stickers and asking whether they wanted to give him any of theirs (“I want to tell you about this kid named Gary. Look, Gary has no stickers. Would you like to give Gary any of your stickers?”). If the child responded yes, the experimenter asked how many stickers they wanted to give and instructed them to put these stickers in front of Gary’s photo. Children were next shown photos of two new children (Jeff and Sam), each of whom had six stickers. One gave five stickers to Gary; the other gave one sticker (e.g., “Now I want to tell you about these two other people. This is Jeff. Jeff has six stickers. Jeff wants to give Gary five of his stickers. This is Sam. Sam has six stickers. Sam wants to give Gary one of his stickers.”). Photographs of the three characters were taken from a database of child faces (LoBue and Thrasher, 2014), and all participants were shown photos of males. Children were then asked to select between these two characters—“Who do you want to be friends with?”—which was adapted from previous work exploring children’s social preferences based on language (e.g., Kinzler et al., 2007).

The procedure for the *No Comparison* condition was identical, with one exception: Here, children were *not* asked if they wanted to give Gary any of their stickers; instead, children simply observed the two characters (Jeff and Sam) giving Gary their respective number of stickers (one and five). In both conditions, we counterbalanced which character (Jeff or Sam) gave their stickers first across children. All sessions were audio-recorded.

### Results

#### Giving Question (Given Only to Children in the Comparison Condition)

Children, on average, gave 2.91 stickers to Gary; every child gave some stickers, with only two children giving five or more stickers and only one child giving as few as one sticker. Thus, the large majority of children (30 of 32) gave fewer stickers to Gary than the generous character and the same as or more than the ungenerous character, creating a situation where children’s own giving compared unfavorably to that of the former and favorably (or comparably, for one child) to that of the latter. Further analyses showed that there were no significant differences in giving between the younger and older halves of our subjects, *t*(30) = 1.14, *p* = 0.26; the mean number of stickers given by the younger 50% of subjects was 2.75 while the mean number of stickers given by the older 50% of subjects was 3.06 stickers.

#### Friend Choice Question (Given to Children in Both Conditions)

Preliminary analyses revealed no significant differences in choices between the younger and older halves of our subjects in the *No Comparison* condition (Fisher’s exact test, *p* = 1.00) and the *Comparison* condition (Fisher’s exact test, *p* = 0.43). Moreover, there were no differences in choices between boys and girls in the *No Comparison* condition (Fisher’s exact test, *p* = 0.52) and the *Comparison* condition (Fisher’s exact test, *p* = 0.70). As a result, ages and gender were combined for all analyses.

**FIGURE 1:**
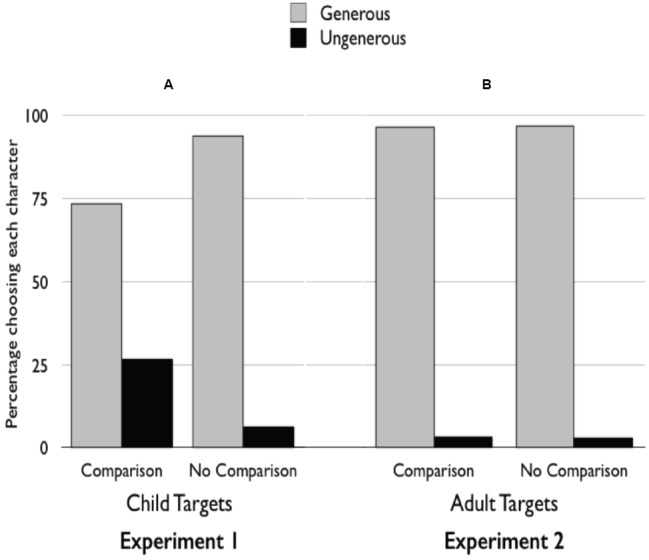
**Percentage of children choosing each character in the ***Comparison*** and ***No Comparison*** conditions in Experiment 1 (A) and Experiment 2 (B)**.

In the *No Comparison* condition, children almost unanimously selected the generous character (30 of 32 children, binomial probability test, *p* < 0.001), showing a strong preference for a generous character over an ungenerous one. For the *Comparison* condition, because we were interested specifically in children’s liking for a generous individual *who showed them up*, we focused on those children who gave fewer stickers than the generous character (30 of 32 children). These subjects also, by and large, preferred the generous character (22 of 30 children, binomial probability test, *p* = 0.016), but this preference was significantly reduced relative to that shown in the *No Comparison* condition (Fisher’s exact test, *p* = 0.04)^1^; see Figure 1A. There was over a *fourfold increase* in children’s choice of the ungenerous character from 6% in the *No Comparison* condition to 26.6% in the *Comparison* condition. Thus, a situation that afforded a comparison of children’s own generosity to that of a generous child significantly reduced their preference for that child, relative to their preference for someone who did not show them up.

## Experiment 2

The results from Experiment 1 suggest that situations that evoke social comparisons decrease children’s liking for extremely generous individuals who outshine children’s own generosity. As a stronger test of our social comparison hypothesis, we examined whether children would reject an extremely generous *adult*. This manipulation decreases the potential for social comparison given that children most strongly compare themselves with others of similar age (e.g., Suls et al., 1978; Dijkstra et al., 2008; Tasimi and Johnson, 2015).

### Participants

Sixty-four children (32 girls; mean age = 9.33 years; range = 8.06–10.42 years) participated in the study. Children were recruited from public schools in the greater New Haven, Connecticut area and tested individually in a quiet room at their elementary school. Parents of participating children gave written informed consent; children also provided oral assent. Data were not gathered on participants’ race/ethnicity; however, children were tested in schools serving communities that were primarily white and middle class. The Human Subjects Committee at Yale University approved all study procedures.

### Procedure

The procedure was identical to Experiment 1 with one exception: Here, we used photos of white male adults (photos taken from Tottenham et al., 2009) to represent Jeff and Sam. Gary, the recipient, remained a child. All participants were shown pictures of male adults to minimize confounds. In particular, if we showed women to girls and men to boys, this would introduce differences in gender attitudes and expectations that are beyond the scope of the current study.

### Results

#### Giving Question (Given Only to Children in the Comparison Condition)

Children, on average, gave 3.12 stickers to Gary; every child gave some stickers, with only three children giving five or more stickers and only two children giving one sticker. Thus, the large majority of children (29 of 32) gave fewer stickers to Gary than the generous character and the same as or more than the ungenerous character, creating a situation where children’s own giving compared unfavorably to that of the former and favorably (or comparably, for two children) to that of the latter. Further analyses showed that there were no significant differences in giving between the younger and older halves of our subjects, *t*(30) = 0, *p* = 1.00; the mean number of stickers given by the younger 50% of subjects was 3.12 stickers and the mean number of stickers given by the older 50% of subjects was 3.12.

#### Friend Choice Question (Given to Children in Both Conditions)

Preliminary analyses revealed no significant differences in choices between the younger and older halves of our subjects in the *No Comparison* condition (Fisher’s exact test, *p* = 1.00) and the *Comparison* condition (Fisher’s exact test, *p* = 1.00). Moreover, there were no differences in choices between boys and girls in the *No Comparison* condition (Fisher’s exact test, *p* = 1.00) and the *Comparison* condition (Fisher’s exact test, *p* = 1.00). As a result, ages and gender were combined for all analyses.

In the *No Comparison* condition, children almost unanimously selected the generous character (31 of 32 children, binomial probability test, *p* < 0.001), showing a strong preference for a generous character over an ungenerous one. For the *Comparison* condition, we focused again on those children in the *Comparison* condition who gave fewer stickers than the generous character (29 of 32 children); these children also showed a strong preference for the generous character (28 of 29 children, binomial probability test, *p* < 0.001). Unlike Experiment 1, children’s social preferences did not differ in the *Comparison* and *No Comparison* conditions (Fisher’s exact test, *p* = 1.00); see Figure 1B.

While there was no difference in children’s responses in the *No Comparison* condition of Experiments 1 and 2 (Fisher’s exact test, *p* = 1.00), their responses in the *Comparison* condition differed significantly (Fisher’s exact test, *p* = 0.026). Thus, children’s rejection of do-gooders in the current investigation seems to occur when they observe generous acts performed by a child, but not by an adult.

## Discussion

Generosity is one of the heavenly virtues, but our results suggest that it may be a mixed blessing when another’s giving outshines one’s own. Although children in the current study reliably preferred a generous to an ungenerous character, this preference decreased considerably when children’s own generosity was inferior to another child’s, but not when it was inferior to that of an adult. These results provide converging evidence, alongside recent studies on adults, documenting the phenomenon of do-gooder derogation. They are also the first (to our knowledge) to show that by middle childhood, social comparison seems to modulate children’s general tendency to prefer individuals who behave generously.

Our findings are also notable because they challenge an alternative explanation for do-gooder derogation, namely that exceptional behavior is aversive because it deviates from the norm. Under this account, any deviation from the norm—whether it is positive or negative—should lead to negative evaluations. In support of this explanation, when adult subjects were asked to provide reasons for expelling extremely generous individuals from their group, many described their generosity as unusual (Parks and Stone, 2010). Additionally, people willingly punish generous individuals, especially when their giving seems atypical compared to other people that gave (Irwin and Horne, 2012). The current study design affords a test of this explanation: Since both characters deviated equally from children’s own level of giving—the modal amount children gave was three stickers—a normative account would not predict a strong preference for the more generous character. Yet, children almost unanimously preferred the more generous character, a preference that was attenuated, but not eliminated, when children’s own generosity was less than that of another child. Future work should therefore investigate the development and conditions under which children do, and do not, use social and cultural norms to reject generous individuals.

While our findings show that situations that evoke social comparisons decrease children’s liking for a generous child, they do not tell us *why* some children preferred a stingy child instead of a generous one. There are at least three distinct interpretations for this finding in the context of our study: (1) the selfish child becomes more attractive; (2) the generous child becomes less attractive; (3) the selfish child becomes more attractive *and* the generous child becomes less attractive. This is an interesting question that future work could examine through measures such as preference *ratings* rather than preference *rankings*. Also, such work could also ask children how they feel after learning about others that gave more or less than themselves. It may be that a selfish individual becomes more attractive because they boost a child’s self-esteem, whereas a generous individual becomes less attractive because they lower a child’s self-esteem. Moreover, it is important that future work also examines whether children’s own giving moderates their social preferences. For example, does a child that gives just one sticker show a stronger aversion toward an extremely generous individual than a child that gives four stickers? The current findings cannot speak to this issue as the vast majority of our subjects gave three stickers; however, this is a promising avenue for future work that could be influenced by a number of factors, including the types of goods being offered (e.g., stickers versus dollars) as well as the type of behavior in question (e.g., sharing versus helping). Additionally, it is worth emphasizing that our studies involved children from largely white and educated households, which raises the question of whether the effect reported here generalizes to other cultures. Following prior research showing that the degree to which people punish high contributors varies substantially across societies (Herrmann et al., 2008), it may be that, in some cultures, children exalt other children who engage in extraordinary acts of giving, even when their own giving may seem inferior in comparison.

Finally, given previous work identifying multiple ways in which moral behavior is rejected (Monin, 2007), it is critical to understand influences beyond social comparison that lead to the rejection of benevolent others. Are we suspicious of extremely generous behaviors and invoke ulterior motives to explain them? Do we anticipate moral reproach from extremely generous others? These questions become important and intriguing given our finding that children’s attraction to generous individuals decreases when another child shows them up.

### Conflict of Interest Statement

The authors declare that the research was conducted in the absence of any commercial or financial relationships that could be construed as a potential conflict of interest.

## Footnotes

1 A strong test of our hypothesis would be to evaluate whether children’s choice of the generous child varies as a function of their own level of giving. Specifically, on our hypothesis, children who gave four or fewer stickers (and were therefore shown up by the generous child) would be expected to select the stingy child more frequently than children who gave five or six stickers (whose giving was equal to or greater than the generous character’s). However, almost *all* children (*N* = 30) gave four or fewer stickers; only two children gave five or more stickers, so no meaningful analysis can be made between the two groups (however, for informational purposes, of these two subjects that gave five stickers, one child chose the generous child and the other chose the stingy child). The vast majority of our subjects (*N* = 22) gave exactly three stickers.
